# Emergency Department Utilization, Hospital Admissions, and Office-Based Physician Visits Among Under-Resourced African American and Latino Older Adults

**DOI:** 10.1007/s40615-021-01211-4

**Published:** 2022-01-10

**Authors:** Sharon Cobb, Mohsen Bazargan, Shervin Assari, Lisa Barkley, Shahrzad Bazargan-Hejazi

**Affiliations:** 1 Mervyn M. Dymally School of Nursing, Charles R. Drew University of Medicine and Science, Los Angeles, CA USA; 2 Department of Family Medicine, Charles R. Drew University of Medicine and Science (CDU), Los Angeles, CA USA; 3 Department of Public Health, CDU, Los Angeles, CA USA; 4 Physician Assistant Program, CDU, Los Angeles, CA USA; 5 Department of Family Medicine, UCLA, Los Angeles, CA USA; 6 Department of Psychiatry, UCLA, Los Angeles, CA USA

**Keywords:** Emergency department utilization, Hospital admissions, Physician visits, African Americans, Latinos, Quality of life, Older adults, Access to medical care

## Abstract

**Objectives:**

This study uses a theoretical model to explore (a) emergency department (ED) utilization, (b) hospital admissions, and (c) office-based physician visits among sample of under-resourced African American and Latino older adults.

**Methods:**

Nine hundred five African American and Latino older adults from an under-resourced urban community of South Los Angeles participated in this study. Data was collected using face-to-face interviews. Poisson and logistic regression analysis were used to estimate the parameters specified in the Andersen behavioral model. Predictors included predisposing factors, defined as demographic and other personal characteristics that influence the likelihood of obtaining care, and enabling factors defined as personal, family, and community resources that support or encourage efforts to access health services.

**Results:**

African American older adults have a greater frequency of hospital admissions, ED, and physician visits than their Latino counterparts. About 25%, 45%, and 59% of the variance of the hospital admissions, ED utilization, and physician visits could be explained by predisposing and enabling characteristics. Lower health-related quality of life was associated with a higher number of hospital admissions, ED, and physician visits. Financial strain and difficulty accessing medical care were associated with a higher number of hospital admissions. Being covered by Medicare and particularly Medi-Cal were positively associated with higher hospital admissions, ED, and physician visits.

**Discussion:**

Compared to African American older adults, Latino older adults show higher utilization of (a) emergency department (ED) utilization, (b) hospital admissions, and (c) office-based physician visits. A wide range of predisposing and enabling factors such as insurance and financial difficulties correlate with some but not other types of health care use. Multi-disciplinary, culturally sensitive, clinic- and community-based interventions are needed to address enabling and predisposing factors that influence ED utilization and hospital admission among African American and Latino older adults in under-resourced communities.

## Introduction

Increasing evidence reveals that emergency department (ED) utilization and hospital admissions are potential disparities among racial and ethnic minority older adults in the USA [1, 2]. Due to multiple chronic conditions and lack of social resources, the process of aging may be accelerated in underserved and under-resourced minority communities, contributing to increased vulnerability and dependence on medical care services, especially for African American and Latino older adults [3–5]. As of 2020, African American and Latino older adults represented 9% and 8% of the U.S. older adult population of 49 million individuals [6]. However, it is estimated that the U.S. population of older racial and ethnic minorities will increase 115% by 2040 [6–8]. About nine out of ten African American older adults are born in the USA compared to four out of ten Latino older adults [8, 9]. Yet, foreign-born Latino older adults may have worse physical and mental health compared to U.S.-born Latino older adults, which may allude to a myriad of migration experiences [10–12]. It is well documented that African American and Latino older adults in the USA suffer from excessive co-morbidities, undiagnosed and uncontrolled medical chronic conditions, and insufficient access to medical care, all of which impact their healthcare utilization, particularly ED utilization and hospital admissions [13–16]. However, there is still limited data on underserved and under-resourced African American and Latino older adults and their usage and interactions with ED utilization and hospital admissions .

Recent data from the Centers for Medicare and Medicaid Services (CMS) revealed that African Americans (73.7%), and Latinos (66.4%) had higher rates of multi-morbidity compared to 55.2% of non-Latino White older adults [17]. Additional data showed 33.7% of African Americans and 19.6% of Latinos reported at least six chronic conditions, whereas only 9.9% of non-Latino White older adults reported this health status [17]. These outcomes suggest that aggressive interventions are needed to decrease existing racial and ethnic health disparities [7]. Reducing racial disparities in chronic care management among older adults requires further exploration of health utilization patterns and factors, including ED utilization, hospital admissions, and office-based visits among this population of older minorities.

National and community-based data have shown that ED utilization by older adults has steadily increased over the previous three decades [18–20]. As a result, ED care is shifting to increase their contribution to medical management care of older adults [21]. During 2014–2017, more than 20% of all ED visits in the USA consisted of older adult patients, with one out of four ED visits resulting in a hospital admission [22]. Specifically, older adults who utilize the ED have distinct patterns of use, leading to increased usage of resources as their age advances [23]. Given that the older adult population is projected to increase for the next few decades, it is likely that older individuals will make up an increasingly larger share of ED visits in the coming years [6, 22].

Moreover, ED visits by under-resourced racial and ethnic minority older adults may affect health outcomes and status. Thus, hospital-based EDs have a critical role in managing the medical care of under-resourced racial and ethnic minority older adults. The 2017 National Hospital Ambulatory Medical Care Survey data (NHAMCS) examined ED data and multiple dimensions of ED care and treatment from 2005 to 2016 among adults [24]. Results revealed a huge racial disparity in ED visits, with 89% of African American respondents utilizing the ED, compared to 40% of Non-Latino Whites and 38% of Latinos. Among older adults, 64% of African Americans visited the ED, compared to 33% of non-Latino Whites [24]. Moreover, race was associated with significant differences in ED treatment and admission rates, which also represent disparities in emergency care. This may be attributed to racial and ethnic minority groups managing more chronic health conditions than non-Latino adults. Specifically, national data shows that African American patients received lower triage scores and higher mortality rates [25]. Another study focused on structural and geographic determinants of ED use in California showed that high rates of ED visits are associated with less access to primary health care [26].

With higher rates of multiple comorbidities and health inequalities, it may be assumed that hospitalization rates remain greater among racial and ethnic minority older adults. However, some large national studies have reported lower hospitalization rates in this group when compared to their non-Latino White counterparts [27, 28]. For example, in a study of 175 million ED visits, Lo and colleagues (2017) documented that African Americans had lower admission rates than Whites (31% vs. 35%) [28]. Similarly, previous NHAMCS data shows racial disparities in hospitalization rates for ED patients with heart failure [27].

National data shows that African Americans and Latinos have high preventable hospitalization rates for major chronic conditions, even after disease prevalence and underlying hospital utilization patterns are considered [29]. Danziger and colleagues (2020) examined the quality of critical care over the last 10 years and revealed that critically ill racial and ethnic minority individuals are disproportionately cared for in racial and ethnic minority-serving hospitals, which have shown significantly less improvement than non-minority-serving hospitals over the last 10 years [30]. Understanding the contributing factors for these disparities may guide interventions to reduce ED hospitalizations for racial and ethnic minority older adults [28].

Additionally, inequities exist for racial and ethnic minority groups in having a primary care provider and usual source of care, utilizing office-based primary care, and specialized health care [31, 32]. In 2016, there were 884 million physician office visits in the USA [33]. Yet, empirical evidence shows different responses at different levels of health across racial and ethnic groups in the use of ambulatory healthcare services, even when stratified by health insurance coverage [34]. For many conditions, increased quality is implied by, and inseparable from, improved access to care [35]. Moreover, African Americans and Latinos may report lower levels of access to appropriate and timely healthcare than their non-Latino White counterparts [36–38]. One of the driving forces for racial and ethnic disparities of office-based care includes a lack of culturally competent educational programs, communication tools, and self-management [39]. Ongoing research is needed to track health service use and access patterns, especially among under-resourced racial and ethnic groups to determine whether existing interventional efforts have reduced long-standing disparities [35].

It is also important to understand the correlates of healthcare utilization when examining racial and ethnic health disparities among the older adult population. This is particularly important due to the paucity of scientific evidence on hospital and ED visits within the Latino older adult population. Evaluating and tracking healthcare utilization, particularly ED visits and hospital admissions, among racial and ethnic minority older adults may help identify subgroups who are at increased risk of poorer physical or mental health and potentially inform the development of effective health-promotion and disease-prevention interventions.

This study uses a theoretical model of health services utilization to examine ED utilization, hospital admissions, and office-based physician visits among a sample of underserved urban African American and Latino older adults. A description of the model, its major components, and its major assumptions follow.

### Theoretical Framework

This study was guided by the Andersen Behavioral Model of Health Care Utilization as its theoretical framework [40]. Within this framework, predictors of healthcare utilization include predisposing characteristics, enabling factors, need-for-care characteristics, and health behaviors. Predisposing characteristics encompass background and sociocultural factors (e.g., gender, age, ethnicity) and health beliefs (e.g., disease knowledge), which can affect individuals’ propensity to use healthcare services. Enabling factors include familial, neighborhood, and community resources and their accessibility to individuals. Examples include access to health insurance and types/levels of social support. Additionally, need-for-care characteristics include health conditions, illnesses, and statuses (perceived and evaluated statuses) that necessitate the individual to access and receive health services. Lastly, health behaviors are comprised of personal health practices, usage of health services, and the process of attaining and receiving medical care [40]. Overall, this model conceptualizes healthcare utilization as the end-product of a complex pattern of interactions among these predictors and was thus deemed to be appropriate for this study.

Our aim was to investigate predisposing and enabling factors that correlate with three indicators of health service use: (a) ED utilization, (b) hospital admissions, and (c) office-based physician visits. In this study, we explored the role of predisposing factors defined as demographic and other personal characteristics that influence the likelihood of obtaining care and enabling factors defined as personal, family, and community resources that support or encourage efforts to access health services. Given the exploratory nature of our study, we did not have a specific hypothesis but centered on generalized theory-based predisposing and enabling factors to be associated with health-service use. Based on the tenets of the Andersen Behavioral Model of Health Care Utilization, we expected differences in these associations with three domains of health service use of focus, namely, (a) ED utilization, (b) hospital admissions, and (c) office-based physician visits. We expect determinants that facilitate office-based physician visits to vary than those relevant to ED use or hospitalization.

## Materials and Methods

Our study sample included 905 older adults that were recruited from senior housing areas, senior community centers, and faith-based organizations. Using convenience and snowball sampling strategies, we recruited from 52 community sites in South Los Angeles. This study’s geographical setting, South Los Angeles, is included in the Service Planning Area 6 (SPA 6) of Los Angeles County, which is recognized as one of the most under-resourced areas in the nation [41]. SPA 6 is disproportionately affected by multiple health disparities, such as having the highest hospitalization rate for heart failure within Los Angeles County. Participants who self-identified as African American or Latino, aged 55 years or older, and were resided in South Los Angeles were eligible for this study. All research instruments, including surveys, were provided in English and Spanish. After providing informed consent, enrolled participants completed face-to-face structured interviews with bilingual research assistants, including those identified as African American and Latins. The Institutional Review Board of XXXXXXX approved the study protocol.

### Survey Instruments

#### Predisposing Characteristics

These variables included age, gender, educational attainment, marital status, and race/ethnicity as the predisposing covariates. Educational attainment was operationalized as a continuous variable, with higher scores indicated more years of education. Marital status was assessed, which was analyzed categorically as either married/lived with a partner or not married/do not live with a partner.

#### Enabling Variables

##### Financial Strain.

Participants were asked, “In the past 12 months, how frequently were you unable to: 1) buy the amount of food your family should have?; 2) buy the clothes you feel your family should have?; 3) pay your rent or mortgage?; 4) pay your monthly bills?; and 5) make ends meet?” Items were on a 5-level response scale ranging from 1 (never) to 5 (always). A total “financial strain” score was calculated by averaging the scores of the five items, resulting in a score ranging from 1 to 5. A higher score was indicative of greater financial difficulty.

##### Accessibility of Medical Care.

Participants were asked, “In the last 12 months: (1) have you needed to see a doctor/healthcare provider but could not see one?; (2) how difficult is it for you to get medical care?; (3) how difficult would it be for you to get a routine physical exam?; (4) how difficult is it for you to visit a doctor when you need medical care?; and (5) how satisfied are you with how available medical care is for you?” Principal component analysis was utilized to identify potential factors, with only one factor being produced that explained almost 50% of the variance. All items had primary loadings over 0.4. A lower score on this index reflects a higher level of perceived difficulty accessing care.

##### Medicare and Medi-Cal.

Participants reported if they had Medicare or Medi-Cal health insurance. Participants were told that Medicare provides health coverage to individuals 65 years of age and older or those with a severe disability regardless of income, whereas Medi-Cal (California’s state-run and funded Medicaid program) provides health coverage to those families with very low income, as well as pregnant women and disabled persons, among others. They were told that Medicare has four parts that cover different things—hospitalization, medically necessary services, supplemental coverage, and prescription drugs.

#### Need-for-Care Characteristics

##### Major Chronic Conditions.

Participants self-reported the following chronic illnesses and conditions that they were managing: high blood pressure/hypertension, diabetes mellitus, heart-related conditions, cancer, stroke, chronic obstructive pulmonary disease (COPD), and sleeping disorders.

#### Health-Related Quality of Life Survey (SF12)

To report quality of life, the 12-item Short Form Health Survey (SF-12) is a validated measure that have been widely utilized to examine the health status of low-income racial and ethnic minority populations [42]. The SF-12 survey includes two subscales on mental and physical functioning, the physical and mental health composite scores (PCS & MCS) [43]. Survey scores range from 0 to 100, where a zero score indicates the lowest level of health and a score of 100 indicates the highest level of health.

##### Smoking Status.

Participants were asked to describe cigarette smoking behaviors. Response options included the following: still smoke, used to smoke, and never smoked. Two dummy variables were created to depict smoking behaviors: (1) past smoker (yes = 1, no = 0), and (2) current smoker (1 = yes, no = 0), whereas (3) never smoker was used as a reference category.

#### Primary Outcomes

##### ED Utilization.

Participants were asked whether they visited the ED within the previous 12 months. To measure, participants noted if they visited the ED for any medical symptoms, traumatic injuries, or unrelated reason. This variable was operationalized as a binary (0/1) variable.

##### Office-Based Physician Visit.

Participants were asked whether they had visited a primary care physician/healthcare provider in the outpatient/clinic setting within the past 12 months.

Hospital admission was examined by asking participants if they had been hospitalized or admitted for inpatient care at a medical facility within the previous 12 months.

### Data Analysis

Our analysis had three parts. The first section was a descriptive analysis of all participants. This descriptive work reported means and standard deviation for continuous measures and frequency and percentages for the categorical variables. Next, we conducted Pearson correlation coefficients, independent *t* test, and ANOVA to examine the bivariate association between each index of healthcare utilization (office-based physician visits, hospital admission, and ED utilization) and all other independent variables. We used multivariate generalized linear models (GLM) with Poisson distribution and log link to examine the independent association of ED utilization and hospital admission with the study variables. However, we employed multiple logistic to examine the role of physician visits. While both ED utilization and hospital admissions had a Poisson distribution, the number of physicians did not; therefore, multiple logistic regression was the appropriate model for physicians’ data. We reported parameter estimate of the association between independent variables and the number of (1) physician visits, (2) hospital admissions, and (3) ED utilization within the last 12 months using odds ratio (OR), and 95% confidence intervals, and *p* values of less than 0.05 were considered significant. Finally, it is not feasible to compute standard *R*^2^ with Poisson or logistic regression models. Instead, we report the pseudo *R*^2^ for GLM and Nagelkerke *R*^2^ for binary logistic regression.

## Results

Table 1 reports the characteristics of the study sample. This study included 740 (82%) African American and 165 (18%) Latino individuals between the ages of 55 and 95 years (mean = 71.50 ± 8.36). Approximately 34% of participants were 75 years of age or older, with 20% self-reported being married or living with a partner. One-third of the sample never completed high school. One out-of-two participants indicated that they never smoked, whereas 31% admitted that stopped smoking, and close to 19% self-identified as current smokers. Regarding health status, we noted the following health conditions/illnesses: diabetes mellitus (38%), hypertension (89%), heart-related conditions (28%), and COPD (27%). The average physical QOL was 40.08, with the average mental QOL at 51.92.

**Table 1:** Characteristics of participants (*n* = 905)

	*N*	%
Gender		
Male	317	35.0
Female	588	65.0
Race/ethnicity		
Latino	165	18.2
Non-Latino African American	740	81.8
Married/partner		
No	726	80.2
Yes	179	19.8
Smoking status		
Never smoker	447	50.1
Former smoker	279	31.2
Current smoker	167	18.7
Medicare		
No	330	36.6
Yes	571	63.4
Medi-Cal		
No	514	57.0
Yes	387	43.0
	Mean	SD
Age	71.50	8.36
Education	11.85	3.32
Financial strains	04.05	1.22
Major chronic conditions	02.08	1.11
Access to medical care	00.00	1.00
Quality of life—physical	40.08	12.15
Quality of life—mental	51.92	11.60
Physician visits (*n*)	5.32	3.21
ED admissions (*n*)	0.75	1.50
Hospital admissions (*n*)	0.71	1.27

### Bivariate Analysis

Table 2 shows bivariate correlations between hospital admissions, ED visits, physician visits, and independent variables. These correlations are based on the Pearson correlation (*r*), the *t* test, and the ANOVA *F* test. The first column of this table shows that ethnicity/race identification of African American, not being married/no partner, a higher level of financial strain, possession of Medicare and Medi-Cal, current-smoking status, a higher number of chronic conditions, and lower level of mental and physical health-related quality of life all correlated with a higher number of physician visits. The second column of Table 2 reveals that being African American, not married/no partner, possession of Medi-Cal, lower level of access to medical care, a higher number of chronic conditions, a higher number of office-based physician visits, and lower levels of health-related quality of life were associated with a higher number of ED utilizations within last 12 months. Finally, the last column of Table 2 shows that being African American, not married/no partner, possession of Medi-Cal and lower level of access to medical care, as well as a higher number of chronic conditions, lower level of health-related quality of life, and a higher number of physician visits within the last 12 months; all were associated with a higher number of hospitalizations.

**Table 2:** Bivariate analysis of healthcare utilization among underserved African American and Latino older adults (*n* = 905)

Independent variables	Health care utilization					
	Physician visits	Sig	Hospital admissions	Sig	ED admission	Sig
	Mean ± SD (Pearson *r*)		Mean ± SD (Pearson *r*)		Mean ± SD (Pearson *r*)	
*Predisposing characteristics*						
Gender						
Male	5.28 ± 3.28	0.757	0.77 ± 1.34	0.784	0.46 ± 1.03	0.412
Female	5.35 ± 3.17		0.74 ± 1.58		0.53 ± 1.38	
Age (year)	(0.019)	0.569	(0.001)	0.977	(r = − 0.009)	0.784
Race/ethnicity						
Latino	4.49 ± 2.78	0.001	0.36 ± 0.98	0.001	0.56 ± 1.03	0.001
African American	5.51 ± 3.27		0.54 ± 1.32		0.79 ± 1.58	
Education (years)	(0.021)	0.523	(0.022)	0.513	(− 0.001)	0.979
Married/live with partner						
No	5.44 ± 3.23	0.022	0.80 ± 1.56	0.030	0.51 ± 1.16	0.954
Yes	4.83 ± 3.07		0.56 ± 1.20		0.50 ± 1.33	
*Enabling characteristics*						
Financial strains	(− 0.077)	0.021	(− 0.019)	0.560	(− 0.130)	0.001
Medicare						
No	4.73 ± 3.11	0.001	0.44 ± 1.14	0.197	0.78 ± 1.41	0.665
Yes	5.66 ± 3.22		0.55 ± 1.34		0.73 ± 1.55	
Medi-Cal						
No	4.90 ± 3.12	0.001	0.43 ± 1.16	0.043	0.61 ± 1.27	0.001
Yes	5.89 ± 3.24		0.61 ± 1.40		0.94 ± 1.75	
Access to medical care (extremely difficult to not at all)	(*r* = − 0.047)	0.163	(*r* = − 0.068)	0.043	(− 0.070)	0.036
*Need for care characteristics*						
Smoking						
Never smoker	5.11 ± 3.21	0.035	0.49 ± 1.33	0.502	0.65 ± 1.44	0.042
Past smoker	5.38 ± 3.04		0.58 ± 1.38		0.77 ± 1.40	
Current smoker	5.86 ± 3.45		0.44 ± 0.92		0.99 ± 1.81	
Major chronic conditions (1–6)	(0.147)	0.001	(0.161)	0.001	(0.168)	0.001
Physical health QoL (low to high)	(− 0.213)	0.001	(− 0.139)	0.001	(− 0.175)	0.001
Mental health QoL (low to high)	(− 0.111)	0.001	(− 0.106)	0.002	(− 0.134)	0.001
Number of physician visits	(0.147)	0.001	(0.161)	0.001	(0.168)	0.001

### Multivariate Analysis

Tables 3, 4, and 5 contain the results from the hierarchical Poisson and logistic regressions. They include the regression coefficients, the ratio of the expected number of visits associated with individuals who differ by one unit on the independent variables, and a pseudo-*R*^2^ coefficient. We entered the predisposing characteristics on the first step of the analysis; the enabling and need-for-care characteristics on the second and third step, respectively.

**Table 3:** Generalized linear models with Poisson distribution and log link: parameter estimate of association between predisposing, enabling, and need-for-care characteristics and number of emergency room utilization *within last 12 months* among underserved Latino and African American adults (*n* = 905)

Independent variable	Model 1			Model 2			Model 3		
	Predisposing characteristics			Predisposing and enabling characteristics			Predisposing, enabling, and need-for-care characteristics		
	OR	95% CI	Sig	OR	95% CI	Sig	OR	95% CI	Sig
*Predisposing characteristics*									
Age (years)	0.981	0.972–0.990	0.000	0.991	0.981–1.002	0.098	0.996	0.985–1.007	0.503
Gender (male vs. female)	0.972	0.828–1.139	0.722	0.974	0.828–1.145	0.746	0.918	0.775–1.089	0.328
Race/ethnicity (Latino vs. AA)	1.646	1.248–2.172	0.000	1.924	1.440–2.571	0.000	1.712	1.256–2.335	0.001
Years of education	0.961	0.935–0.989	0.006	0.978	0.949–1.008	0.155	0.976	0.946–1.007	0.122
Married/partner (no vs. yes)	0.738	0.592–0.921	0.007	0.852	0.680–1.068	0.164	0.836	0.664–1.051	0.125
*Enabling characteristics*									
Financial strains (always to never)				0.852	0.797–0.912	0.000	0.936	0.868–1.009	0.083
Medicare (no vs. yes)				1.002	0.839–1.196	0.982	0.949	0.789–1.140	0.575
Medi-Cal (no vs. yes)				1.419	1.204–1.672	0.000	1.198	1.012–1.418	0.036
Access to medical care				0.947	0.879–1.020	0.152	0.982	0.907–1.063	0.653
*Need for care characteristics*									
Current smoker (ref. nonsmoker)							1.025	0.817–1.418	0.830
Past smoker (ref. nonsmoker)							0.935	0.774–1.063	0.484
Major chronic conditions (1–6)							1.213	1.130–1.302	0.000
Physical health QoL (low to high)							0.983	0.976–0.990	0.000
Mental health QoL (low to high)							0.989	0.982–0.995	0.001
Number of physician visits							1.054	1.028–1.080	0.000
Model likelihood ratio chi-square	40.148			94.566			204.809		
Sig	0.000			0.000			0.000		
Pseudo *R*-squared	0.0161			0.0386			0.0851

**Table 4:** Generalized linear models with Poisson distribution and log link: parameter estimate of association between predisposing, enabling, and need-for-care characteristics and number of hospital admissions within last 12 months among underserved Latino and African American adults (*n* = 905)

Independent variable	Model 1			Model 2			Model 3		
	Predisposing characteristics			Predisposing and enabling characteristics			Predisposing, enabling, and need-for-care characteristics		
	OR	95% CI	Sig	OR	95% CI	Sig	OR	95% CI	Sig
*Predisposing characteristics*									
Age (years)	0.997	0.986–1.009	0.658	0.997	0.985–1.010	0.677	0.999	0.986–1.012	0.902
Gender (male vs. female)	1.185	0.970–1.447	0.096	1.198	0.980–1.466	0.078	1.044	0.844–1.291	0.692
Race/ethnicity (Latino vs. AA)	1.686	1.200–2.367	0.003	1.847	1.302–2.622	0.001	1.557	1.076–2.253	0.019
Years of education	0.983	0.948–1.018	0.333	0.996	0.960–1.034	0.839	1.005	0.967–1.045	0.797
Married/partner (no vs. yes)	1.088	0.854–1.387	0.495	1.207	0.942–1.545	0.137	1.161	0.899–1.501	0.253
*Enabling characteristics*									
Financial strains (always to never)				1.002	0.918–1.094	0.964	1.172	1.065–1.291	0.001
Medicare (no vs. yes)				1.176	0.944–1.466	0.148	1.034	0.822–1.300	0.776
Medical (no vs. yes)				1.427	1.171–1.740	0.000	1.208	0.984–1.483	0.071
Access to medical care				0.852	0.782–0.928	0.0000	0.877	0.799–0.962	0.006
*Need for care characteristics*									
Current smoker (ref. nonsmoker)							0.689	0.510–0.932	0.016
Past smoker (ref. nonsmoker)							1.046	0.842–1.300	0.684
Major chronic conditions (1–6)							1.226	1.127–1.334	0.000
Physical health QoL (low to high)							0.977	0.969–0.986	0.000
Mental health QoL (low to high)							0.979	0.971–0.987	0.000
Number of physician visits							1.077	1.045–1.110	0.000
Model likelihood ratio chi-square	12.737			46.754			184.726		
Sig	0.026			0.000			0.000		
Pseudo *R*-squared	0.0062			0.0229			0.0920

**Table 5:** Multivariate logistic regression: parameter estimate of association between predisposing, enabling, and need-for-care characteristics and number of office-based physician visits within last 12 months among underserved Latino and African American adults (*n* = 905)

Independent variable	Model 1			Model 2			Model 3		
	Predisposing characteristics			Predisposing and enabling characteristics			Predisposing, enabling, and need-for-care characteristics		
	OR	95% CI	Sig	OR	95% CI	Sig	OR	95% CI	Sig
*Predisposing characteristics*									
Age (years)	0.989	0.973–1.006	0.196	0.990	0.972–1.008	0.283	1.000	0.980–1.020	0.964
Gender (male vs. female)				1.047	0.780–1.405	0.761	1.069	0.778–1.468	0.681
Race/ethnicity (Latino vs. AA)				2.370	1.441–3.899	0.001	1.995	1.172–3.396	0.011
Years of education				0.974	0.923–1.029	0.350	0.973	0.919–1.030	0.341
Married/partner (no vs. yes)				0.758	0.518–1.109	0.153	0.728	0.488–1.086	0.120
*Enabling characteristics*									
Financial strains (always to never)				0.848	0.743–0.968	0.014	0.977	0.845–1.131	0.757
Medicare (no vs. yes)				1.616	1.174–2.225	0.003	1.720	1.229–2.406	0.002
Medi-Cal (no vs. yes)				1.596	1.188–2.144	0.002	1.366	1.003–1.860	0.048
Access to medical care				0.928	0.801–1.074	0.316	0.959	0.821–1.120	0.597
*Need for care characteristics*									
Current smoker (ref. nonsmoker)							1.401	0.905–2.171	0.131
Past smoker (ref. nonsmoker)	1.020	0.765–1.359	0.895				1.258	0.897–1.765	0.183
Major chronic conditions (1–6)	2.169	1.344–3.503	0.002				1.171	1.022–1.342	0.023
Physical health QoL (low to high)	0.946	0.898–0.997	0.038				0.974	0.961–0.987	0.000
Mental health QoL (low to high)	0.655	0.453–0.947	0.025				0.981	0.968–0.994	0.005
− 2 log likelihood	1191			1152			1090		
Sig	0.000			0.000			0.000		
Nagelkerke *R*-squared	0.031			0.080			0.136		

#### ED Utilization

Table 3 reports the results of three GLM with Poisson distribution and log link for ED utilization. The first model shows that predisposing characteristics accounted for a marginally significant amount of variance in the outcome (Nagelkerke pseudo-*R*^2^ = 0.0161; *P* < 0.001), representing approximately 2% of the variance. All predisposing variables except gender were significant predictors of ED utilization. The second model in Table 3 shows that both predisposing and enabling characteristics accounted for 3.86% of the variance. However, the third model revealed that introducing the need-for-care variables raised the explained variance to almost 9%. Examining the three models in Table 3 shows that the predisposing variables were responsible for close to 19% (0.0161/0.0851 = 0.189) of the total variance explained with the full model, whereas both predisposing and enabling characteristics shared 45% (0.0386/0.0851 = 0.454) of the total variance. The final model in Table 3 shows that a higher number of ED visits within the last 12 months was associated with being African American, possession of Medi-Cal, higher number of major chronic conditions, lower levels of both physical and mental health-related quality of life, and a higher number of office-based physician visits, controlling for all other variables.

#### Hospital Admissions

Table 4 reports the results of three GLM with Poisson distribution and log link for hospital admissions within the last 12 months. The predisposing alone, predisposing and enabling variables together, and full model (that includes predisposing, enabling, and need-for-care) accounted for less 1%, 2.3%, and 9.2% of the variance of hospital admissions. Therefore, the predisposing variables were responsible for only 6.7% of the total variance explained with the full model, whereas both predisposing and enabling characteristics shared 24.9% of the total variance. A higher number of hospital admissions was associated with identification as African American (compared to Latino), more financial strain, greater difficulty accessing medical care, a higher number of chronic conditions, a lower level of physical and mental health-related quality of life, and a higher number office-based physician visits, adjusting for other variables.

#### Physician Visits

Table 5 reports the results of three multiple binary regression models for office-based physician visits within the last 12 months. The first model shows the association between predisposing variables and physician visits. The second model included both predisposing and enabling variables, whereas the last model includes all variables. Nagelkerke *R*^2^ examination shows that 22.8% of total variance was explained by predisposing variables and 58.9% of total variance by a combination of predisposing and enabling characteristics together. The final model confirms that higher number of physician visits was associated with being African American, possession of Medicare and Medi-Cal, a higher number of chronic conditions, and lower levels of both physical and mental health-related quality of life.

## Discussion

This study found that 28% of African American and 23% of Latino older adults had at least one hospital admission within a 1-year period. Additionally, 41% of African Americans and 32% of Latinos in this study visited an ED at least once during the last year. Finally, 32% of Latinos and 46% of African Americans had at least five office-based physician visits within the same 1-year period. Our multivariate analyses introduced a range of predisposing, enabling, and need-for-care determinants of ED utilization. Examination of our data shows that 25%, 45%, and 59% of the total variance for hospital admissions, ED utilization, and office-based physician visits were accounted for by predisposing and enabling characteristics, respectively. Despite introducing five measures of the need-for-care (major chronic conditions, smoking behaviors, and both mental and physical health-related quality of life), predisposing and enabling characteristics are still significantly larger contributors, particularly for office-based physician visits and ED utilization. The impact of the need-for-care characteristics may differ for hospital admission and ED utilization, because hospital admission and ED utilization are commonly nondiscretionary behaviors, as opposed to office-based physician visits.

The dominance of predisposing and enabling characteristics on ED utilization among our sample of underserved racial and ethnic minority older adults, challenges the assumption that ED use may be the result of nondiscretionary behavior [20]. The number of older adults presenting to the ED is increasing, with evidence showing that older adults are visiting the ED multiple times during a calendar year [44]. Therefore, we propose two explanations for the lack of stronger impact of need-for-care characteristics on ED visits. First, if an ED visit substitutes a routine office visit; then, it is expected that the enabling and predisposing variables will show a stronger impact. Second, it is plausible that more refined instrumentation of the need-for-care characteristics is needed to challenge the dominance of predisposing and enabling factors. For example, the inclusion of acute illness and accidental injuries may increase the explanatory power of need-for-care characteristics [45].

### Race/Ethnicity and Office-Based Physician Visits, Hospital Admissions, ED Utilization

Latino older adults had a decreased utilization rate of all types of healthcare compared to their African American counterparts. Previous studies have revealed disparities in healthcare service use among Latinos relative to other ethnic groups [46, 47]. Moreover, Latinos in the USA may be more likely to delay the needed care for chronic conditions than other ethnic groups and less likely to utilize the healthcare system for preventative and emergent care [48]. Latino older adults may be more likely to utilize Social Security and Supplemental Security Income (SSI) than employer-sponsored retirement plans as their main source of income [49, 50]. Yet, older undocumented Latino adults are a vulnerable population that report multiple health issues [48]. A recent study revealed that immigrants faced various barriers to care in the USA, and their lack of prior healthcare access further discouraged health care utilization and compromised their medical care experiences after migration [51, 52]. The high cost of healthcare and limited access to care may result in poorer outcomes among undocumented older adults [53]. Furthermore, the exclusion of undocumented Latino older adults from receiving full Medi-Cal benefits may increase the health vulnerability of the family [54]. This can affect their spending for healthcare expenditures, as their income is limited to governmental provisions. These systemic inequities in access to long-term care may lead to lower quality of care [55]. Additionally, this population may be disproportionately affected due to socioeconomic disadvantage and systemic racism, which may precipitate their usage of the ED.

### Enabling Characteristic and Healthcare Utilization

Overall, our data showed that for both bivariate and multivariate analysis, enrollment with Medi-Cal leads to a higher number of hospital admissions, ED visits, and office-based physician visits. Similarly, at the multivariate level, enrollment with Medicare was strongly associated with office-based physician visits. Analyzing longitudinal data from Health and Retirement Study, Tavares and colleague (2020) revealed that racial and ethnic minority older adults in poorer communities had a higher proportional risk of utilizing Medicaid than their White counterparts [56]. They suggest that Medicaid is currently serving the most vulnerable older citizens, and any reduction of benefits would be detrimental to these individuals [56]. However, using nationally representative data from the Health and Retirement Study, Bakk and Cadet (2018) documented that African American and Latino older adults are less likely to be aware of the Medicaid Part D Low-Income Subsidy, which typically covers the cost of medications [57]. In an additional study, individuals in low-income housing who visited a primary care provider in the office setting had lower odds of visiting the ED [58]. These findings have implications for primary care practice. Since 80% of participants in this study visited an office-based physician at least three times, there may be opportunities to screen for and address the predisposing and enabling characteristics in the ambulatory care setting and prevent ED visits and hospitalizations.

In addition, our data shows that at the bivariate level, difficulty accessing medical care was associated with a higher number of hospital and ED admissions. At the multivariate level, difficulty accessing medical care was associated with a higher number of hospital admissions among our sample of African American and Latino older adults. Moreover, a subsample of Latino older adults who used ED services within last 12 months were asked, “How often do you use the emergency room because it is more convenient?” More than 43% responded that “sometimes, most of the time, or always” they use the ED because it is more convenient than visiting a regular healthcare physician. One recent study shows that patients living in medically underserved areas and areas with lower spatial access to primary care clinics had higher odds of preventable ED use [59]. Other studies have documented that low versus high continuity of care is associated with greater risk of frequent ED utilization [60]. These findings provide more support for the notion that many underserved elderly racial and ethnic minorities who have difficulty accessing primary care providers and resort to EDs as a substitute for regular sources of medical care are potentially being hospitalized with poorer outcomes. Creative solutions are necessary to improve the value and ensure the quality of emergency care delivered to vulnerable older adults while more fully addressing their complex underlying physical, social, cognitive, and situational needs [61].

### Need-for-Care Characteristics and Healthcare Utilization

We documented that health-related quality of life (HR-QoL) was associated with ED visits and hospital admissions. Controlling for other relevant variables, including predisposing and enabling characteristics, and the number of chronic conditions, HR-QoL showed a strong association with hospital admissions, ED utilization, and office-based physician visits. It is important to mention that HR-QoL (SF-12) is a self-reported outcome measure assessing the impact of health on an individual’s everyday life and its domains includes functional limitations, pain, vitality, general health perceptions, general psychological distress, and well-being [43]. Representing both perceived and functional and mental aspects of life, our data show that lower HR-QoL was linked to higher healthcare utilization among our sample of African American and Latino older adults. Indeed, it has been documented that poor HR-QoL is associated with first ED visit in older adults [62]. Latinos, on the other hand, accumulate chronic disease at a faster rate relative to White adults [63]. Both African American and Latino older adults reported poorer health than their White counterparts [64, 65]. Specifically, there is sufficient evidence showing that both African American and Latino adults are more likely to rate their health as fair/poor [66] and these racial inequities in self-rated health increase in magnitude as age increases. Furthermore, examination of 9 years of data from the population-based Behavioral Risk Factor Surveillance System (BRFSS) shows that HR-QoL and self-rated health worsened in most demographic groups, including African Americans and Latino middle-age and older adults [67]. HR-QoL continues to offer an irreplaceable entry point into perspectives on life and health, particularly as a large group of middle-aged persons approach old age with historically poor self-rated health status [68]. Therefore, the HR-QoL of underserved and under-resourced African American and Latino older adults should be routinely tracked in order to document the effectiveness of the nation’s overall health gain against 2030 Healthy People objectives [67]. This information provides opportunities for targeted interventions to reduce such disparities among older underserved adults [69].

Moreover, older adults who access the ED is linked to greater number of chronic conditions and less office-based physician visits. A recent study found high ED utilization among Latino older adults, primarily for their chronic conditions, including circulatory and respiratory conditions [70]. Data from the Health and Retirement Study (HRS), a longitudinal nationally representative study of middle-aged and older adults in the USA, shows that middle-aged African American adults start at a higher level of chronic disease burden and develop multi-morbidity at an earlier age, on average, than their non-Latino White counterparts. Certain need-for-care characteristics, including certain diagnoses and poor general health can lead to ED recidivism among older adults [71]. Evidence-based strategies and policies centered on illness management in both the primary care and ED setting can lead to better appropriation of resources and re-orientation of older adults to proper levels of care.

Our data showed a significant bivariate association between all types of healthcare utilization and smoking status, indicating that current smokers were more likely to be admitted to the hospital, utilize the emergency room, and visit a physician/healthcare provider. Also, in multivariate analysis, controlling for all other relevant variables, a higher number of hospital admissions was found among current smokers, participants with lower level of physical and mental health-related quality of life, and self-reported binge drinking. It is established that cigarette smoking exacerbates the severity of chronic illnesses and impairs successful symptoms management [72–74]. Knowing that racial and ethnic minority middle-aged and older adults are at greater risk for chronic disease morbidity than their non-Latino White counterparts, effective interventional studies must target racial and ethnic minority older smokers with co-morbidities [75]. Another recent study found that a large majority of middle-aged and older smokers continued to smoke after being diagnosed with a major chronic disease [76, 77]. Also, African American older adults are more likely than their White counterparts to change their smoking behavior once diagnosed with chronic conditions. Quiñones and colleagues suggest, for racial and ethnic minorities in middle and late life, the onset of a diagnosis may be an actionable or “teachable moment” for smokers to change [75]. Screening and providing early intervention tailored to the level of use and risk are needed to address the health need of this group [78].

### Study Limitations

The limitations of this study include the non-random convenient sample and cross-sectional design of the study, which limits its generalizability. Because of the cross-sectional design of our study, we cannot make any causal inferences. Second, we did not have access to objective medical histories and records for the participants; therefore, we relied on self-report of chronic conditions. Third, our Latino sample size was lower than that for African American older adults. As this study was a face-to-face interview, even though we had planned to collect additional survey from Latinos, we halted data collection because of COVID-19. Additionally, participant data focused on country of origin and years lived in the USA was not collected. Yet, we did ask participants for their preferred language and provided survey assistance in English and Spanish. Finally, although these results were statistically significant, their clinical and public health significance needs further investigation. Despite these limitations, the results contribute to the current understanding of correlates of healthcare utilization among underserved older African American and Latino adults and potentially contribute to the design of culturally sensitive interventional studies to reduce health disparities among this segment of our population.

## Conclusion

Our study documented that 4 out of 10 underserved Latino and African American older adults visited the ED within last 12 months. In addition, the dominance of predisposing and enabling characteristics on ED utilization challenge the assumption that emergency use is the result of nondiscretionary behavior, which is another strong indication of health disparities among older racial and ethnic minority adults in the USA. Our data shows that there may be opportunities to screen for and address the social determinants (predisposing and enabling characteristics) in the ambulatory care setting and prevent non-urgent ER visits as well as reduce preventable hospital admissions.

Strong association between HR-QoL and ED utilization, hospital admissions, and office-based physician visits are another indication that HR-QoL continues to offer an irreplaceable entry point into perspectives on life and health among racial and ethnic minority older adults. Therefore, the HR-QoL of underserved African American and Latino older adults should be routinely tracked by primary care physicians.

## Data Availability

The data sets used and analyzed in the current study are available from the corresponding author for collaborative studies.

## Code Availability

N/A.
